# A Hypomorphic *PALB2* Allele Gives Rise to an Unusual Form of FA-N Associated with Lymphoid Tumour Development

**DOI:** 10.1371/journal.pgen.1005945

**Published:** 2016-03-18

**Authors:** Philip J. Byrd, Grant. S. Stewart, Anna Smith, Charlotte Eaton, Alexander J. Taylor, Chloe Guy, Ieva Eringyte, Peggy Fooks, James I. Last, Robert Horsley, Antony W. Oliver, Dragana Janic, Lidija Dokmanovic, Tatjana Stankovic, A. Malcolm R. Taylor

**Affiliations:** 1 Institute of Cancer and Genomic Sciences, University of Birmingham, Birmingham, United Kingdom; 2 Genome Damage and Stability Centre, University of Sussex, Brighton, United Kingdom; 3 University Children’s Hospital, School of Medicine University of Belgrade, Belgrade, Serbia; St Jude Children's Research Hospital, UNITED STATES

## Abstract

Patients with biallelic truncating mutations in *PALB2* have a severe form of Fanconi anaemia (FA-N), with a predisposition for developing embryonal-type tumours in infancy. Here we describe two unusual patients from a single family, carrying biallelic *PALB2* mutations, one truncating, c.1676_1677delAAinsG;(p.Gln559ArgfsTer2), and the second, c.2586+1G>A; p.Thr839_Lys862del resulting in an in frame skip of exon 6 (24 amino acids). Strikingly, the affected individuals did not exhibit the severe developmental defects typical of FA-N patients and initially presented with B cell non-Hodgkin lymphoma. The expressed p.Thr839_Lys862del mutant PALB2 protein retained the ability to interact with BRCA2, previously unreported in FA-N patients. There was also a large increased chromosomal radiosensitivity following irradiation in G2 and increased sensitivity to mitomycin C. Although patient cells were unable to form Rad51 foci following exposure to either DNA damaging agent, U2OS cells, in which the mutant PALB2 with in frame skip of exon 6 was induced, did show recruitment of Rad51 to foci following damage. We conclude that a very mild form of FA-N exists arising from a hypomorphic *PALB2* allele.

## Author Summary

PALB2 is a protein that creates a molecular bridge that promotes the recruitment of Homologous Recombination Repair (HRR) proteins BRCA1, BRCA2 and Rad51 to sites of DNA damage. Cells with functional loss of PALB2 show a defect in HRR and are characterized by an increased number of spontaneous chromosome breaks and hypersensitivity to cross-linking agents. Typically, inherited mutations in *PALB2* are associated with a severe Fanconi Anaemia phenotype. Here we describe for the first time the effect of biallelic mutations of *PALB2* in which one hypomorphic allele resulted in a low level of expression of the mutant PALB2 protein with some retained function, as shown by its interaction with BRCA2, as well as its ability to facilitate Rad51 focus formation following damage. This resulted in a considerably milder clinical phenotype with increased longevity compared with biallelic PALB2 null patients and an altered tumour spectrum towards development of lymphoid malignancies.

## Introduction

BRCA2, PALB2, BRCA1, Rad51 and the Rad51 paralogs form the principal constituents of the homologous recombination (HR) machinery utilised by the cell not only to repair deleterious DNA lesions, such as DNA double strand breaks (DSBs) in an error-free manner, but also to stabilise, protect and restart damaged replication forks. PALB2 [1] functions as a molecular bridge by simultaneously binding both BRCA1 and BRCA2 via its N-terminal coiled-coil domain and C-terminal WD repeats respectively [2–4]. It has been proposed that BRCA1 plays a role in targeting the BRCA2-Rad51 recombination complex to sites of DNA damage directly through its ability to bind PALB2 [3]. However, since PALB2 and Rad51 foci are not completely ablated in the absence of BRCA1 and PALB2 has been shown to bind chromatin directly through its ChAM domain [5], it is likely that PALB2 acts as an essential conduit linking the pro-resection, anti-recombinogenic and the cell cycle checkpoint activities of the various known BRCA1 containing complexes (typified by the presence of RAP80, MERIT40, Abraxas, BRCC36/45, CtIP or FANCJ) with the recombination machinery [6].

It has been known for some time that heterozygosity for a germline mutation in either *BRCA1* or *BRCA2* strongly predispose women to the development of breast and/or ovarian cancer. More recently, heterozygosity for a germline mutation in *PALB2* [7], *RAD51C* [8] and *RAD51D* [9] have also been associated with an increased risk of developing breast and ovarian cancer. These observations highlight a critical role for the HR pathway in protecting against cellular transformation, particularly of hormone-responsive, epithelial cells that have a high proliferative capacity. Interestingly, however, it has been shown that biallelic, null germline mutations in *BRCA2* [10–14] and *PALB2* [15–17], hypomorphic mutations in *BRCA1* [18,19] and *Rad51C* [20], or in the case of *RAD51* [21] a dominant mutation, all give rise to a spectrum of clinical symptoms that have features of Fanconi Anaemia (FA). Unlike Fanconi anaemia patients with mutations of one of the core complex Fanconi proteins, those with inherited mutations in the HR machinery, including *PALB2* mutant patients, typically display severe developmental abnormalities, such as microcephaly, growth retardation, intellectual impairment as well as skeletal abnormalities. These patients also commonly exhibit kidney malformations, microphthalmia, skin hypo/hyper-pigmentation, hypoplastic thumbs and gonadal dysgenesis, although unlike typical FA patients, bone marrow failure is almost never observed. Cells derived from these patients are almost invariably hyper-sensitive to DNA cross-linking agents, e.g. mitomycin C (MMC) and diepoxybutane, as well as ionising radiation (IR), consistent with a defect in homologous recombination. Given the clinical similarity between FA and the consequences of mutations in HR genes, patients with biallelic mutations in *BRCA2*, *PALB2*, *BRCA1*, *RAD51C* and the RAD51 dominant mutation have been designated FA-D1, FA-N, FA-S, FA-O and FA-R respectively. The severity of the defects exhibited by HR-defective patients serves to emphasise the importance of the recombination pathway in dealing with DSBs and damaged replication forks that occur naturally with fairly high frequency during development.

In contrast to the consequences of mono-allelic mutation of these genes in carriers, biallelic null mutations predispose patients to embryonal tumours, e.g. Wilms’ tumour, neuroblastoma, medulloblastoma and AML in infancy. The different spectrum of tumours that develops in the FA-like patients, compared with carriers heterozygous for a germline mutation, probably arises as a consequence of inactivating HR early in embryonal cells versus inactivation in an already differentiated cell type.

Given the rarity of patients with biallelic mutations in genes involved in the HR machinery, the range of the clinical symptoms displayed by the affected individuals is limited. Indeed, all hitherto described FA-N patients have two truncating *PALB2* mutations and a very similar, severe clinical presentation. Here we describe a family with two *PALB2* mutations where the affected individuals presented with few of the clinical features typically exhibited by FA-N patients. Unlike FA-N individuals who present in infancy, patients in this study presented at an older age and did not exhibit most of the developmental abnormalities associated with this FA complementation group. Interestingly, although the affected patients developed tumours at a relatively early age, these were B cell lymphomas rather than embryonal tumours. We demonstrate that the PALB2 protein produced from one of the mutant alleles carried by these patients retains both its N- and C-terminal domain required to interact with BRCA1 and BRCA2 respectively. We also show that this mutant PALB2 preserves some function compared with previously identified patient-associated mutant PALB2 proteins and allows low level recruitment of Rad51 foci at sites of DNA damage. We propose that the presence of this mutant protein accounts for the milder MMC hyper-sensitivity exhibited by cells from these patients and the lack of defining clinical characteristics. Lastly, the identification of this family implicates *PALB2* as a potential suppressor of lymphoid tumourigenesis and raises the possibility that somatic mutations in this gene may contribute to the development of sporadic lymphomas.

## Results

### Patients

A 12 year old girl (II-4) was admitted to hospital in August 2008 with enlarged cervical lymph nodes and enlarged tonsils. The histopathology of the lymph node biopsy revealed B cell non Hodgkin lymphoma, stage II. The tumour exhibited a CD20+, CD79a+, CD 10+, immunophenotype, Bcl-2 focal positivity, Ki-67 >90% positivity, whereas other markers were negative including CD3, CD45RO, CD15, CD30, ALK and EMA.

The patient was subsequently treated according to B NHL BFM 2004 protocol (arm R2: element A twice—dexamethasone, VCR, ARA-C, etoposide, MTX, IFO; element B twice—dexamethasone, VCR, DOXO, MTX, cyclophosphamide) until November 2008 without any episode of WHO Grade III or IV toxicity. Since then, she has entered and remained in complete clinical remission. She is now aged 19 years.

Notably, the patient did not have typical features of Fanconi anemia such as radial aplasia, aplastic anaemia or malformation of the kidneys. Renal echosonography as well as cardiac US were normal. Rather, she showed a slight degree of learning difficulty with problematic speech and pronunciation. However, in similarity with Fanconi anaemia height and weight were both below the 3^rd^ centile for age. She also had a Mongol spot at the sacral area, a pigmented nevus at the right gluteus and dysmorphic facies. Peripheral blood cytogenetic analysis showed a normal karyotype (46, XX), although numerous spontaneous chromatid and isochromatid breaks were observed. Serum AFP was slightly elevated (35.4 μg/L; normal range: 0–13.4 μg/L) and immunoglobulin assay showed elevated IgM at 5.03 g/L. No mutations in the *TP53* gene were detected.

A younger sister (now aged 15 years) (II-5) was also successfully treated for non-Hodgkin lymphoma at age 3.5 years. She had normal CBC, elevated AFP (34.4 μg/L) and normal immunoglobulin levels including IgM. The peripheral blood karyotype obtained from this sibling was also normal. Physical examination showed height and weight both below the 3^rd^ centile, epicanthus, café au lait spot on the left shoulder (4x2 cm) and another larger café au lait spot (>10 cm diameter) on the left hip. Notably, this child had learning difficulty and speech impairment.

**Fig 1:**
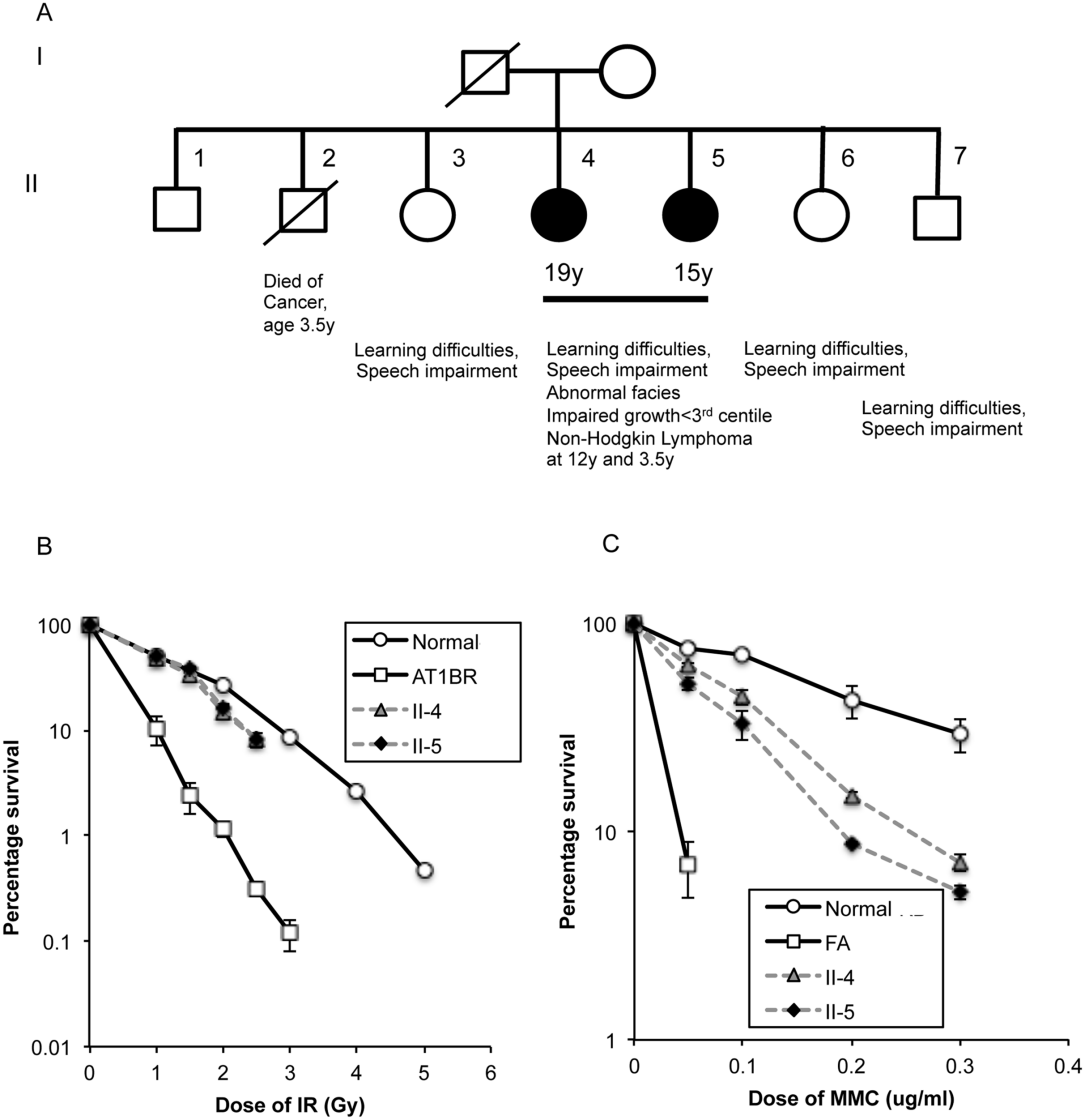
Clinical and cellular features of affected siblings. A) Pedigree of family showing affected siblings. It is not known whether sibling II-2 also had the same syndrome as II-4 and II-5. B) Colony forming assay following exposure of fibroblasts to ^137^Cs γ-rays, C) Colony forming assay following exposure of fibroblasts to Mitomycin C. The proportion (%) of surviving colonies was plotted against dose. The assay was repeated at least three times for each cell strain. Error bars show the s.e.m of survival at each dose.

In August 2008 the family history revealed 5 additional siblings: The oldest brother (II-1) was aged 21 years and healthy, whereas the second brother (II-2) died at age of 3.5 years due to a bone cancer of unknown origin. The oldest sister (aged 15 years) (II-3) and siblings II-6 (aged 6 years) and II-7 (aged 4 years) also showed learning difficulty and speech impairment (Fig 1).

### Unusual sensitivity to ionizing radiation and DNA crosslinking agents

The increased spontaneous chromosome breakage observed in blood lymphocytes from the affected patients (II-4 and II-5) was suggestive of a possible underlying DNA repair deficiency. To investigate this, we assessed the repair capacity of cells derived from one of the affected patients following exposure to IR (using both G2 chromosomal breakage analysis and colony survival assays) and also the DNA cross-linking agent MMC, (using both chromosomal breakage analysis and colony survival assays). Lymphocytes from the affected patient exhibited a high level of chromosome breakage, notably triradials and quadriradials, in response to MMC, similar to the level in two of the control Fanconi patients of unknown complementation group. Strikingly, a very high level of damage in response to IR was seen, higher than the level observed in A-T lymphocytes, indicative of a severe DNA repair defect (Table 1).

**Table 1:** Chromosomal damage in lymphocytes from PALB2 patient II-5 and controls.

Total number of Cells	Number of Rings	Number of Dicentrics	Number of Fragments	Number of Chromatid gaps	Number of Chromatid Breaks	Number of Tri-radials	Number of Quadri-radials	Number of Multi-radials
(1) Patient blood—untreated								
50	0	2	2	8	1	0	0	0
(2) Following exposure to 0.1μgml^-1^ mitomycin C for 4h and cultured to 48h *Patient II-5*								
50	1	2	2	27	20	8	8	3
*Normal control*								
50	0	0	0	0	0	0	0	0
*Three typical Fanconi anaemia patients*								
50	0	0	0	22	11	4	5	0
50	0	0	0	26	21	10	10	0
50	0	0	0	16	19	0	1	0
(3) Following exposure to 1.0 Gray γ-rays@ G2 *Patient II-5*								
50	1	3	4	81	54	29	18	9
*Normal control*								
50	0	0	0	6	0	0	0	0
*Two classical ataxia telangiectasia patients (positive control)*								
50	0	0	0	65	18	1	1	0
50	0	0	0	49	29	1	2	0

Interestingly, in contrast to the results obtained from the chromosomal breakage analysis, fibroblasts from both affected sisters had a normal sensitivity to IR as judged by colony survival and an intermediate sensitivity to MMC, when compared to a fibroblast cell line derived from a typical FA patient of unknown complementation group (although FA-A is the most common)(Fig 1). The apparent disparity between the two different IR assays can be explained by the fact that the IR induced chromosomal breakage is a consequence of unrepaired DNA damage occurring in G2-phase, whereas colony survival assays with skin fibroblasts take into account repair across the entire cell cycle. Given that approximately 85% of all DSBs, irrespective of cell cycle phase, are repaired by NHEJ, a normal survival response to IR, increased G2 chromosomal radiosensitivity and decreased MMC survival indicated a defect in HR.

**Fig 2:**
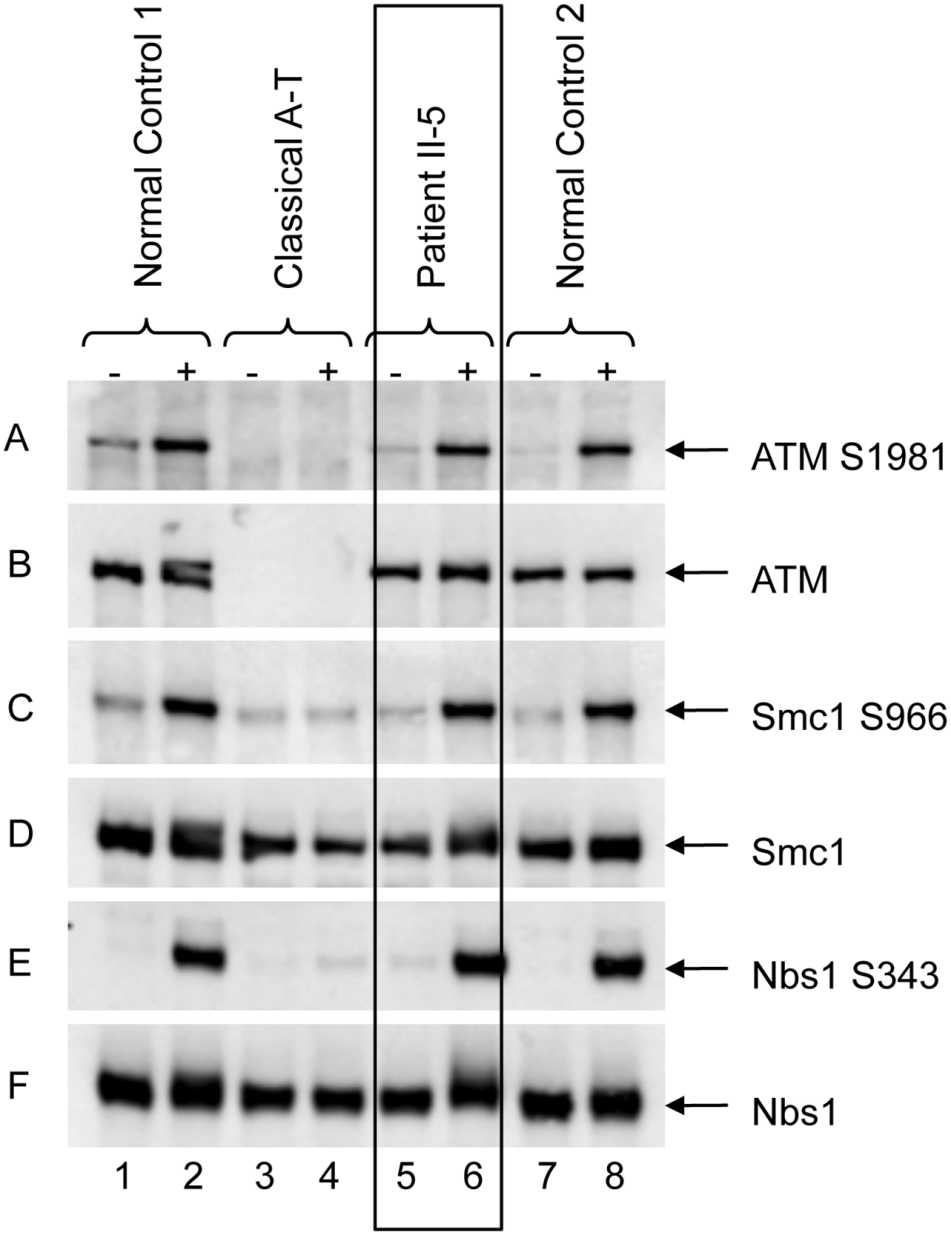
Normal ATM signaling in patient cells. ATM from the lymphoblastoid cell line of II-5 is able to autophosphorylate (panel A), shows a normal level (panel B, lanes 5 & 6) as well as normal signalling as indicated by phosphorylation of ATM targets Smc1Ser966 (panel C) and NbnSer343 (panel E), as is the case for the normal control (lanes 1 & 2). Lanes 3 & 4 show the A-T control, where there is no ATM (panel B) and no evidence of ATM signalling. Panels D & F show the total levels of Smc1 and Nbn respectively.

Since mutations in the Mre11-Rad50-Nbn (MRN) complex give rise to a chromosomal hypersensitivity to both IR and MMC and, at least in the case of NBS patients predisposition to developing lymphoid malignancies, such as B cell lymphomas, we monitored the activation of ATM in response to DSBs using Western blotting coupled with phospho-antibodies to known substrates of ATM. Notably, the IR-induced phosphorylation of ATM (Ser-1981) as well as that of Smc1 (Ser-966) and Nbn (Ser-343), all of which are known to be reduced in the absence of a functional MRN complex, were completely normal in an LCL derived from patient II-5 (Fig 2). Similar results were obtained using fibroblasts derived from both patients II-4 and II-5 (S1 Fig). Since ATM could be efficiently activated in cells from both affected patients and the levels of Nbn were unaltered, this indicated that it was unlikely that the underlying DNA repair defect could be accounted for by a deficiency in the MRN complex.

### Abnormal IRIF focus formation

Given that our preliminary analysis of cells derived from the two affected sisters suggested an underlying HR defect, we next determined whether cells from these patients exhibited any problems recruiting specific DNA repair proteins to sites of DSBs or DNA cross-links. At early time points following exposure to a low dose of IR, fibroblasts from patients II-4 and II-5 were able to relocalise MDC1, 53BP1, BRCA1, FANCD2 and RPA2 to sites of DSBs marked by γH2AX foci, in a manner similar to a normal fibroblast cell line. Comparable results were obtained when the patient cells were exposed to low dose MMC (S2 Fig). However, in keeping with an underlying DNA repair deficiency, both patients’ fibroblasts displayed residual foci at late times post-irradiation (S2 Fig).

**Fig 3:**
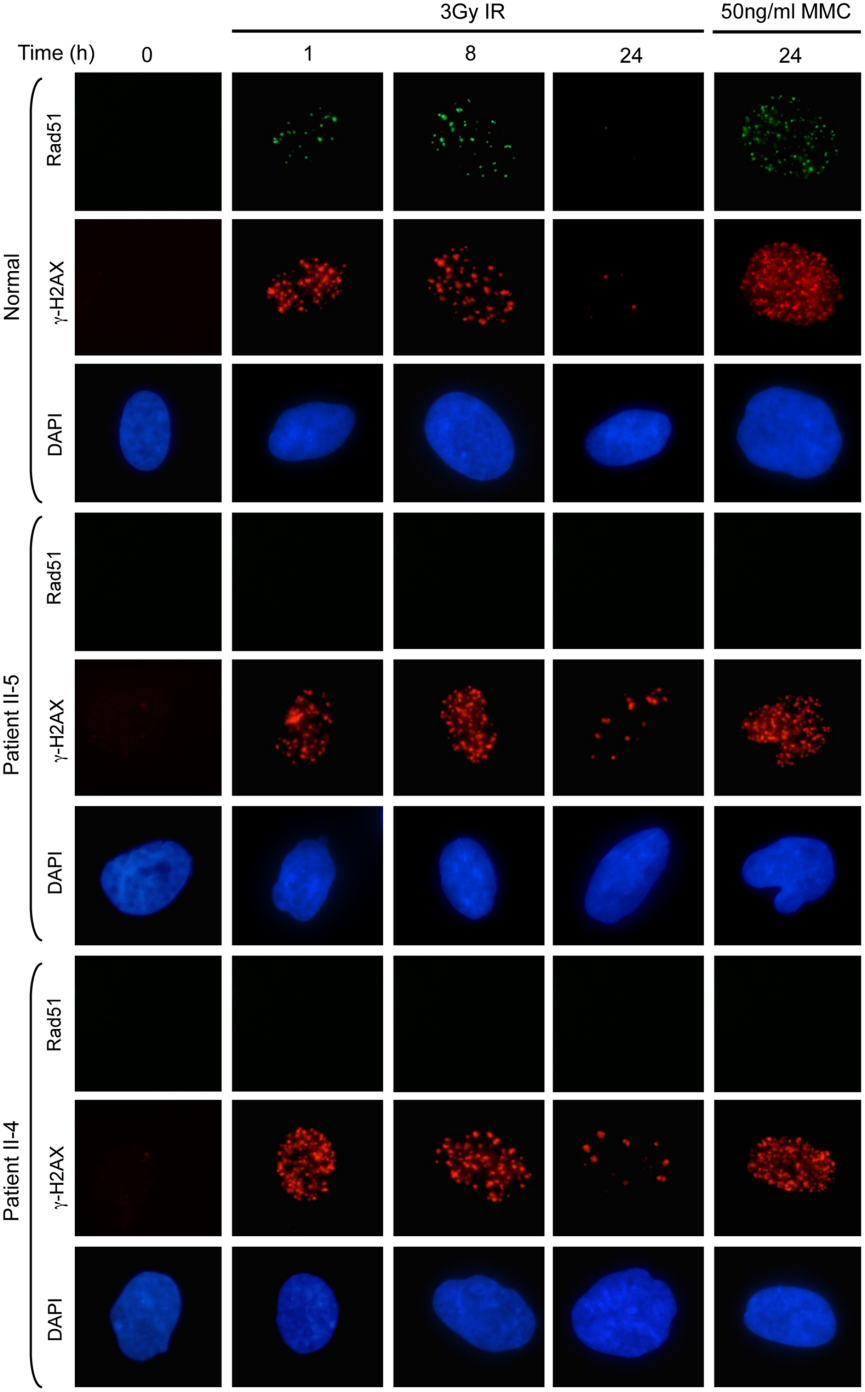
Absence of Rad51 foci in fibroblasts from patients II-4 & II-5. Following exposure to either 3Gy IR or 50ngml^-1^ mitomycin C no Rad51 foci were detected in fibroblasts from II-4 and II-5. γH2AX foci indicate the presence of DNA DSB. The residual foci at 24h following IR are consistent with a DNA repair deficiency.

In stark contrast to our findings assessing the relocalisation of MDC1, 53BP1, BRCA1, FANCD2 and RPA2, fibroblasts from both affected patients completely failed to recruit Rad51 to sites of DNA damage induced by either IR or MMC (Fig 3). In light of the fact that Rad51 foci formation was not completely abolished in the absence of BRCA1 and that cells from our affected patients could still efficiently relocalise BRCA1, this suggested that the underlying genetic defect in our unusual DNA repair deficient family most likely arose as a consequence of mutations in *PALB2*, *BRCA2* or *RAD51*.

### Protein and Mutation analysis in cells from family members

**Fig 4:**
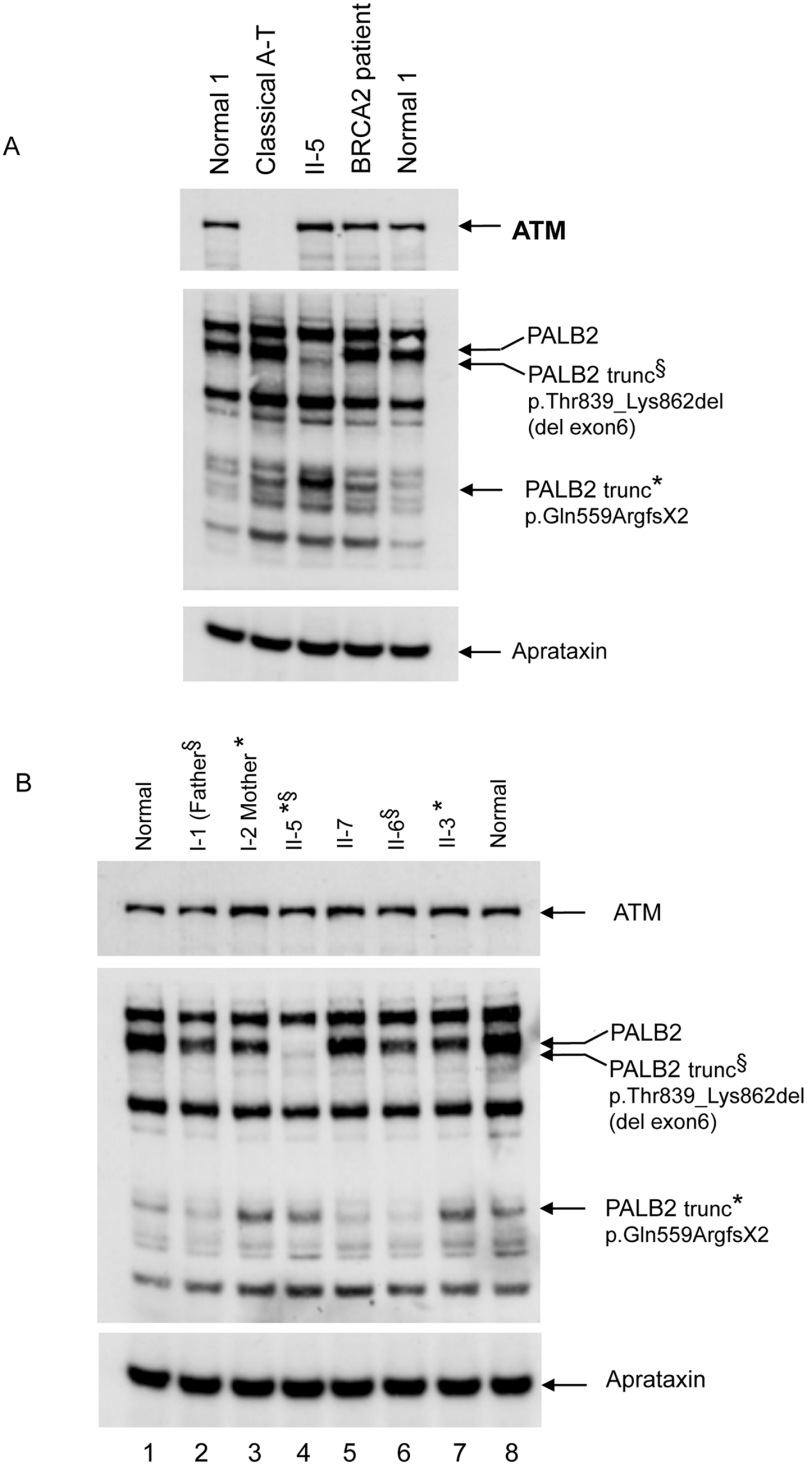
Analysis of expression of PALB2 protein in a family with previously unrecognised FA-N patients. A) Western blot showing loss of full length PALB2 in the affected patient II-5 (Ab 301-246A)(lane 3). However, there is the presence of a faint signal for a slightly smaller protein, probably corresponding to the exon six deletion mutation. Also present in the patient lane is a much smaller band at 90kDa. Lane 4 is lysate of cells from a FA-D1 (biallelic mutation of BRCA2) patient. B) All relatives of the patient II-5 show bands for WT PALB2. Also found in the lanes for the mother (I-2) and sibling II-3, (lanes 3 and 7) is the smaller dense 90kD protein band consistent with I-2 and II-3 carrying the c.1676_1677delAAinsG mutation. In addition, a less dense band of the same size, 90kD, appears as a consequence of the antibody cross reacting with another protein. This cross reacting band is visible in the normal control (both left and right), I-1, II-7 and II-6. The father carries the c.2586+1G>A, although the exon 6 deleted protein cannot be seen against the background of the normal full length protein from his WT allele.

Based on the inability of the affected patient cells to form Rad51 foci in response to DNA damage, we utilised Western blotting to assess the levels of BRCA1, BRCA2, PALB2 and Rad51 in cells from affected patient II-5. Whilst BRCA1 and Rad51 were readily detectable in the affected patient tested compared to normal cells, full length PALB2 was completely absent and the stability of BRCA2 significantly reduced (S3 Fig) presumably due to the low level of stabilising mutant PALB2 protein. Interestingly, two faint lower molecular weight bands detected with the PALB2 antibody were present in cells from patient II-5 indicating the presence of expressed truncated mutant protein (Fig 4A and 4B).

**Fig 5:**
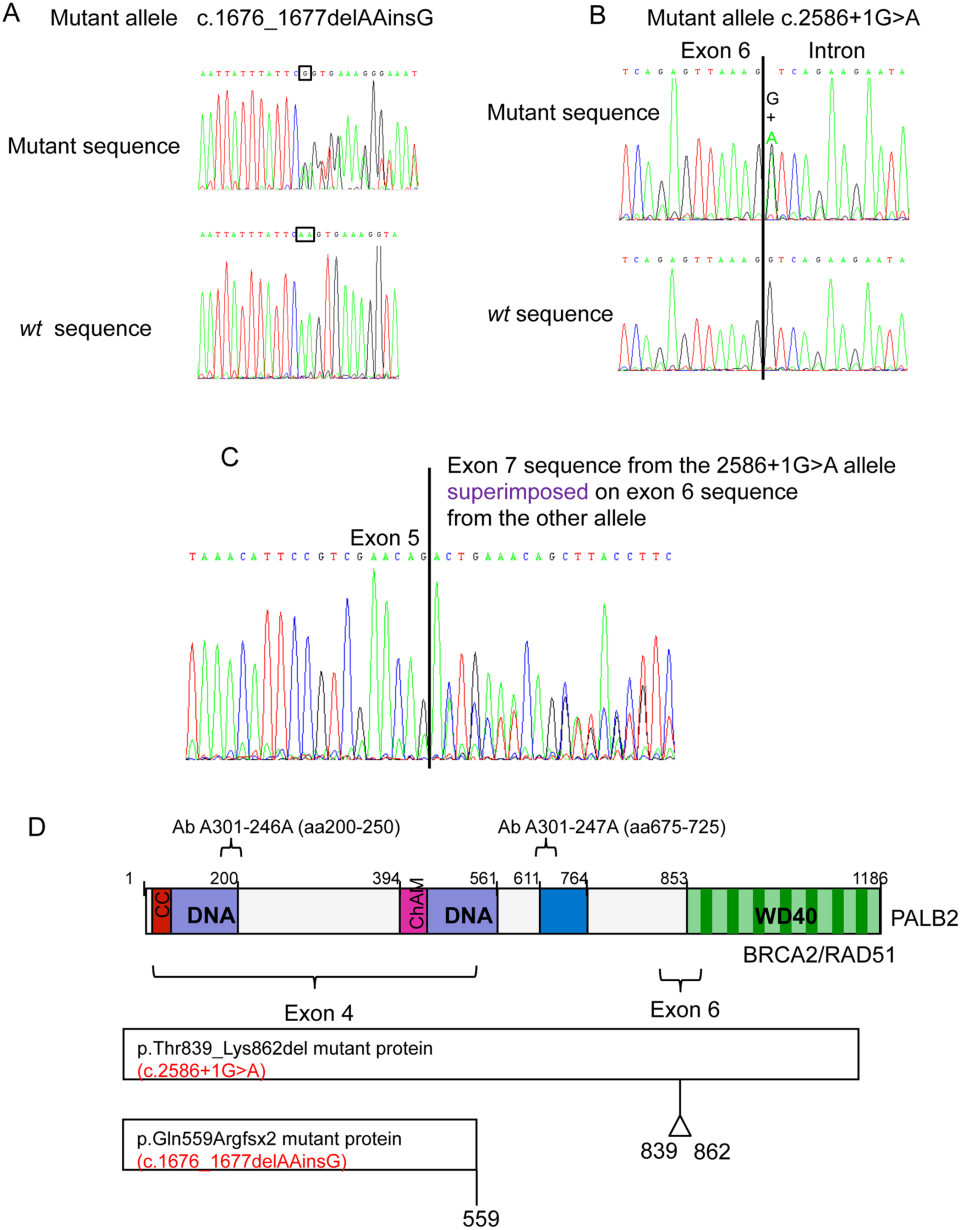
*PALB2* mutations in the affected family. Electropherograms of *PALB2* mutations in DNA in the affected siblings. A) Top panel, c.1676_1677delAAinsG in genomic DNA; lower panel, *wt* sequence at that position. B) Top panel mutant c.2586+1G>A in genomic DNA; lower panel, *wt* sequence at that position. C) cDNA sequencing showing that the c.2586+1G>A mutation causes exon 6 to be omitted during RNA splicing. D) Diagramatic representation of the PALB2 protein and predicted effects of mutations. Diagram of PALB2 showing the epitopes to which the two anti-PALB2 antibodies bind and also the locations of the mutations in the patients. Note that because the epitope for the PALB2 antibody 246A is in exon 4, the exon revertant protein (Exon4delRev) deletion mutant cannot be detected with this antibody. Similarly, because the Y551Ter and Q559RfsTer2 mutants truncate at residues 551 and 559 respectively, they do not possess the epitopes for the 247A antibody. Only wild type and T839_K862del exon six deletion PALB2 are detectable with both antibodies.

Following sequencing of the *PALB2* gene in both sisters, we identified two deleterious mutations: c.1676_1677delAAinsG; (p.Gln559ArgfsTer2) in exon 4 and c.2586+1G>A; (p.Thr839_Lys862del), a base substitution in the first base of intron 6 that affected the donor splice site resulting in an in frame skip of exon 6 (24 amino acids) (Fig 5A–5D). Further analysis of these mutations in other family members revealed that the mother and sibling II-3 inherited the c.1676_1677delAAinsG; (p.Gln559ArgfsTer2)(p.Q559RfsTer2) mutation, whereas the father and sibling II-6 inherited the c.2586+1G>A; (p.Thr839_Lys862del)(p.T839_K862del) mutation and sibling II-7 carried two normal alleles. Mutations were not detected in BRCA2 or RAD51 in II-4 and II-5.

Consistent with the identified mutations, the high and low molecular weight proteins detected in cells from patient II-5 with the PALB2 antibody corresponded to the approximate sizes of the p.Thr839_Lys862del and p.Gln559ArgfsTer2 mutant PALB2 proteins respectively (Fig 4). The smaller protein, at about 90kD, was the same size as another protein that cross-reacted with this antibody.

Interestingly, the N-terminal coiled coil domain (aa9-44) of PALB2 that has been shown to interact with BRCA1 [3,4] was retained in both mutant proteins. In contrast, the C-terminal WD-40 repeat domain of PALB2 that is essential for its interaction with BRCA2 was only preserved in the p.Thr839_Lys862del mutant protein. It was, therefore, speculated that the mild clinical presentation of the disease in affected siblings, together with the absence of embryonal tumours, could be attributed to residual function of the exon 6 deletion mutant PALB2 protein.

### Functional analysis of mutant PALB2 proteins

Remarkably, the two *PALB2* mutations (T839_K862del and Q559RfsTer2) identified in our atypical FA-N family were similar to two *PALB2* mutations (Y551Ter) and an in frame revertant (Exon4delRev) with both the N- and C-terminal domains that was derived by Xia et al, [15] from the EUFA1341 cell line. In keeping with the position of known binding sites on the PALB2 protein, the Y551Ter mutant was unable to interact with BRCA2 and as such could not complement the MMC hypersensitivity or Rad51 foci defect of the EUFA1341 cell line. In contrast, the in frame Exon4delRev PALB2 retained its ability to bind to BRCA2 and could complement cellular defects exhibited by the EUFA1341 cell line [15].

In order to examine the capability of our two different patient mutant PALB2 proteins to interact with BRCA2, isogenic Flp-In/T-Rex U2OS cells were generated that inducibly expressed FLAG tagged WT PALB2, the individual *PALB2* mutations identified in our FANCN family and the individual mutations described by Xia et al, [15]. An siRNA sequence directed against the 3’ UTR of the *PALB2* mRNA (not present in the exogenous *PALB2* open reading frame) was used to deplete endogenous PALB2 protein to prevent the presence of this WT protein interfering with our interaction studies.

**Fig 6:**
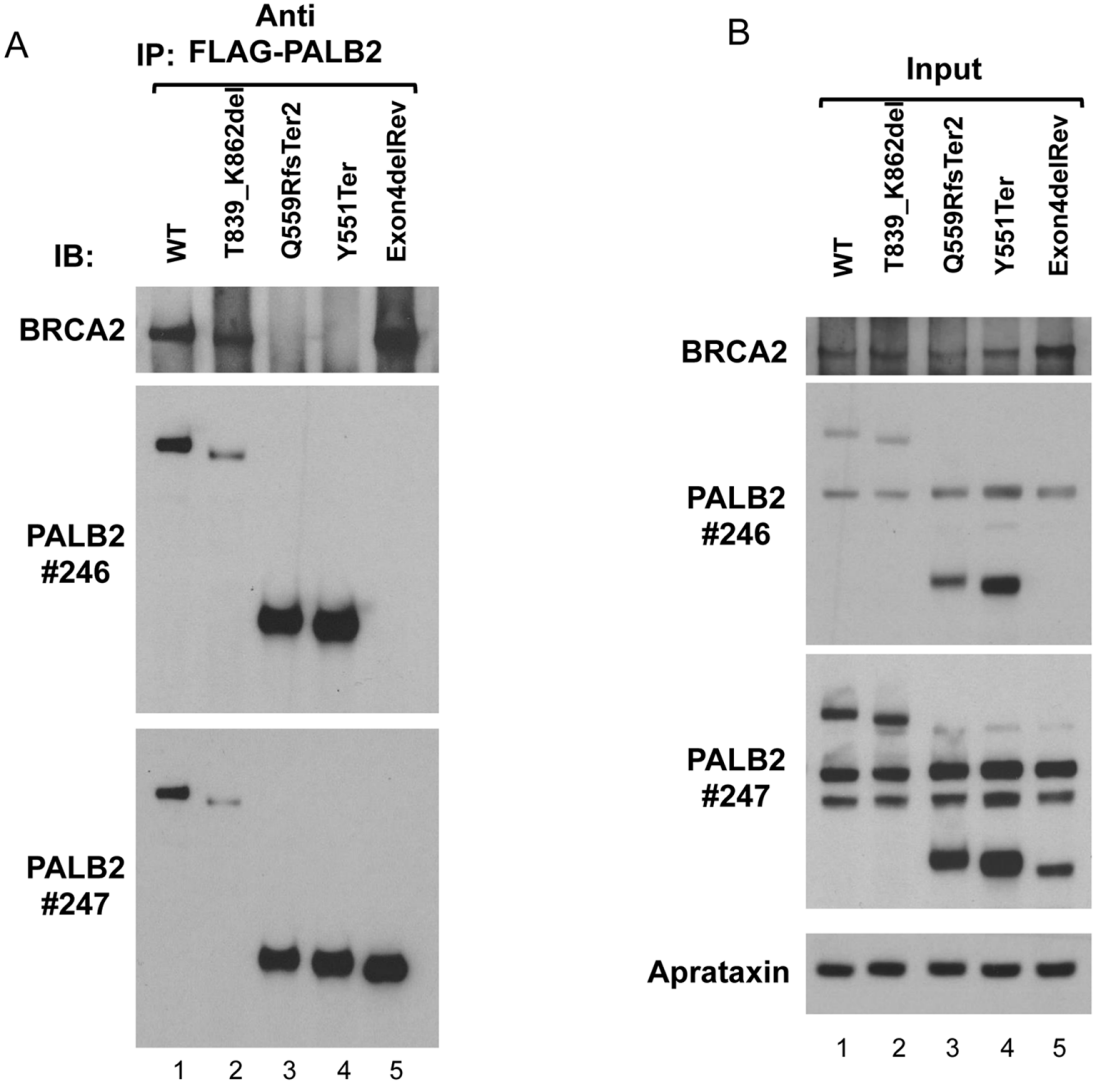
Co-immunoprecipitation of BRCA2 with mutant PALB2 proteins. A) Cultures of U2OS cell lines that inducibly express FLAG-tagged WT or mutant PALB2 proteins were transfected with PALB2 siRNA and induced to express the tagged PALB2 proteins by treatment with doxycycline. Samples were incubated with anti-FLAG antibody and immunoprecipitates electrophoresed and blotted serially with mouse anti-BRCA2 (top panel), rabbit anti-PALB2 246A antibody (middle panel) and then rabbit anti-PALB2 247A antibody (bottom panel). 246A antibody was not stripped before blotting with 247A. A band approximating to 460kDa and detected with anti-BRCA2 antibody was immunoprecipitated with FLAG-tagged WT, T839_K862del (exon 6 deleted) and PALB2 revertant (Exon4delRev) proteins, in those samples that were incubated with anti-FLAG antibody. Anti-PALB2 antibodies identified FLAG-tagged WT protein at ~170kDa as expected (lane 1), with the exon 6 deleted protein appearing to be slightly smaller (lane 2). The FLAG-tagged Q559RfsTer2 and Y551Ter proteins were found at their expected sizes (middle panel). The FLAG-tagged PALB2 revertant protein (Exon4delRev) was evident with Ab 247A in bottom panel. B) Samples of the NETN lysates were taken as ‘input’ samples before immunoprecipitation, and 5% of total lysate immunoprecipitated was subjected to PAGE and Western blotting. The filters were blotted serially with different antibodies as in A. These blots show BRCA2 in all samples before immunoprecipitation. Anti-PALB2 antibodies identify FLAG-tagged PALB2 protein in all WT and mutant expressing cell lines. The aprataxin protein indicates the similarity of loading.

Following siRNA-mediated depletion of endogenous PALB2 protein, the induced FLAG-tagged exogenous PALB2 was immunoprecipitated with an anti-FLAG antibody and the presence of BRCA2 verified by Western blotting. Consistent with previous observations by Xia et al, [15] WT PALB2 and the revertant lacking exon 4 (Exon4delRev), but not the Y551Ter mutant, were able to robustly co-immunoprecipitate BRCA2 (Fig 6A). In a similar fashion, despite being expressed at low levels, the p.T839_K862del mutant PALB2 protein, but not the C-terminally truncated mutant (Q559RfsTer2), could interact with BRCA2 (Fig 6A). Taken together, these data are consistent with the notion that the exon 6 deleted p.T839_K862del PALB2 mutant allele lacking the 9 most N-terminal amino acids of the WD40 repeat region still interacts with BRCA2 and, therefore, possesses some residual activity.

**Fig 7:**
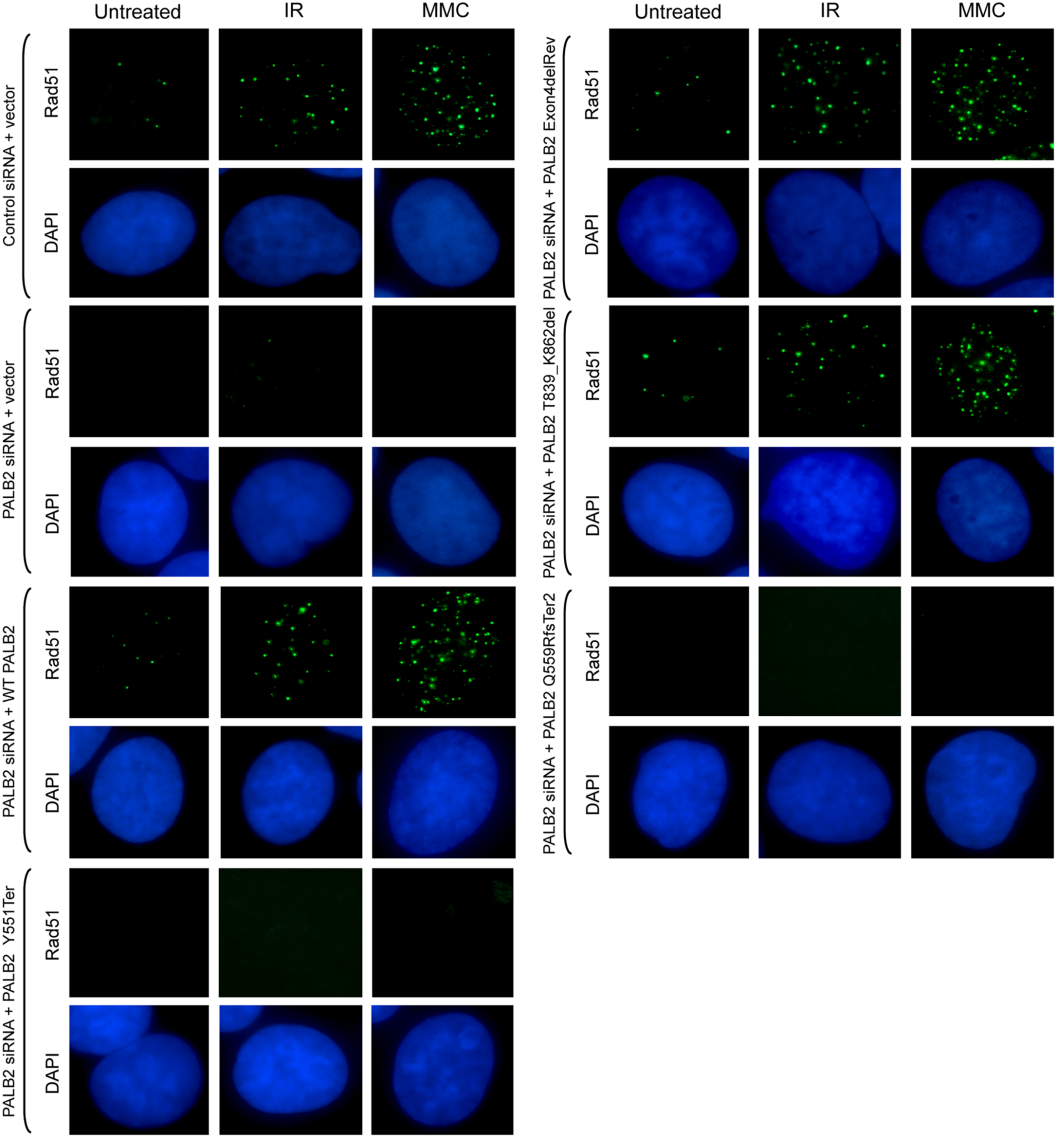
Recruitment of Rad51 foci to sites of damage in U2OS cells expressing FLAG-tagged exon 6 deleted p.T839_K862del mutant PALB2. Recruitment of Rad51 foci to sites of damage in U2OS cells that expressed FLAG-tagged WT, revertant PALB2 lacking exon 4 (Exon4delRev) or exon 6 deleted T839_K862del mutant PALB2, but not in cells expressing Y551Ter (present in the EUFA1341 cell line) or the Q559RfsTer2 PALB2, following depletion of endogenous PALB2 and MMC or IR exposure.

We, subsequently, investigated the activity of the p.T839_K862del mutant PALB2, by carrying out a complementation assay examining the localisation of Rad51 foci in the different U2OS cell lines that expressed either FLAG-tagged WT or mutant PALB2 proteins (S4 Fig). U2OS cells were plated on to coverslips and following siRNA-mediated depletion of endogenous PALB2 protein and induction of the FLAG-tagged exogenous PALB2 the location of Rad51 was determined. Expression of the Y551Ter (present in the EUFA1341 cell line) and the Q559RfsTer2 PALB2 protein from our patient did not permit recruitment of Rad51 foci to the sites of damage following MMC or IR treatment. This was in contrast to induction of the revertant PALB2 lacking exon 4 (Exon4delRev), as well as the exon 6 deleted p.T839_K862del mutant PALB2 in our patient, where recruitment of Rad51 foci following damage was seen in both instances (Fig 7). Therefore, the exon 6 deleted p.T839_K862del mutant PALB2 can, indeed, allow Rad51 recruitment to the sites of damage and the reason for the absence of Rad51 foci in patient cells is related to the low level of expression of this mutant PALB2.

### Prediction of the effect of loss of PALB2 exon 6 on the structure of PALB2

**Fig 8:**
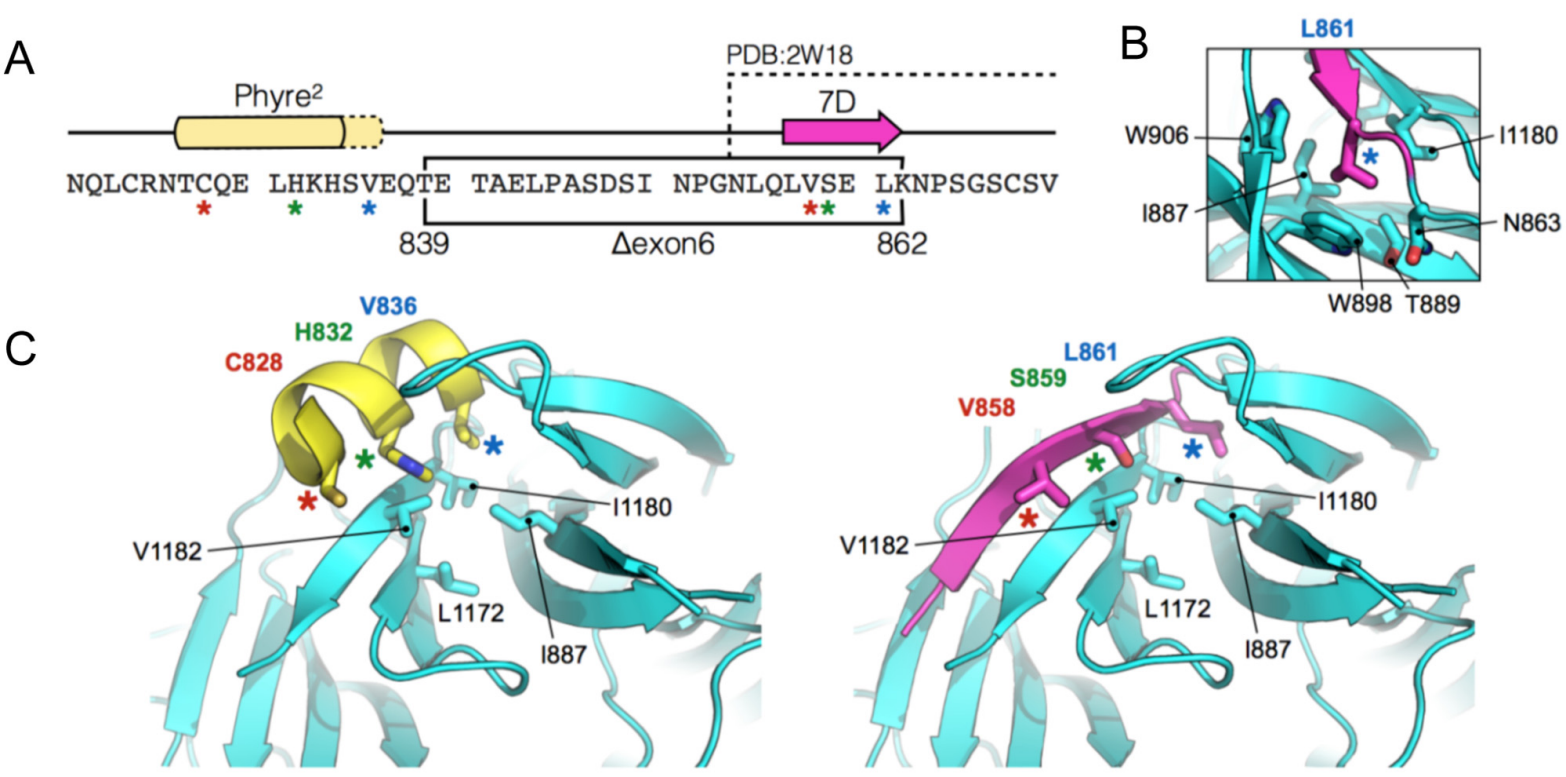
Suggested model of the mutant PALB2 WD40-repeat. A) Amino acid sequence of the region immediately upstream of the PALB2 WD40-repeat. The in-frame deletion, created by the skipping of exon 6, is indicated by the black box. The position of beta-strand 7D and the upstream region of helical propensity are indicated by the magenta arrow and yellow box respectively. The amino acids visible in the X-ray crystal structure of the wild-type PALB2 WD40-repeat (PDB: 2W18) are also indicated. B) In the wild-type protein Leu861 of beta-strand 7D sits in a small hydrophobic pocket lined by the indicated amino acids. C) Molecular cartoons showing the N-terminal part of the WD40-repeat (cyan). (Left) the labelled amino acids (coloured asterisks), on one face of the upstream helical element (yellow), resemble structurally and spatially, those of the 7D beta-strand (Right, magenta).

PALB2 contains a seven-bladed beta-propeller (WD40-repeat) at its C-terminus [22]. Blade 7 of this repeat is formed by C-terminal beta-strands 7A, 7B and 7C, which are ‘zipped’ back to the first N-terminal strand (7D) in order to complete and seal the protein fold. A PALB2 Y1183Ter mutation, which resulted in loss of the 4 most C-terminal residues (strand 7C), is predicted to lead to incomplete folding and instability of the mutant protein [22]. Indeed, no truncated PALB2 protein was detected in patient cell lysates harbouring this mutation [16]. Interestingly, in the patients we describe here, amino acid residues 839–862 are deleted from the protein, which includes the entirety of beta-strand 7D (857–862: LVSELK) (Fig 8A).

Using sequence-threading and manual modelling strategies [23, PyMOL Molecular Graphics System, Version 1.7.4 Schrödinger, LLC] we were able to generate a model for the mutant form of the protein, and compare this to the X-ray crystal structure of the wild-type WD40-repeat (PDB: 2W18). Loss of beta-strand 7D could be expected to lead to destabilisation of the protein fold, as several hydrogen bonds between the component beta-strands that form Blade 7 would not be made. However, this particular region of the protein is also hydrophobic in nature, and the side-chains of beta-strand 7D also contribute to stability of the fold via van der Waals interactions. In particular, the side chain of Leu861 (strand 7D) is buried in a small hydrophobic pocket, lined by residues Asn863, Ile887, Thr889, Trp898, Trp906 and Ile1180 (Fig 8B).

Secondary structure predictions [23] indicate helical propensity for the amino acid sequence immediately upstream of strand 7D. The in-frame deletion of exon 6, would lead to a direct fusion of this helical region to the start of the WD40-repeat (i.e. replacing beta-strand 7D). Side chains of Cys828, His832 and Val836 protrude from one face of the modelled helix, and can be positioned such that they resemble in part, structurally and spatially, those of beta-strand 7D (Val858, Ser859 and Leu861 (Fig 8C). Moreover, Val836 is positioned to interact with the hydrophobic pocket—normally occupied by Leu861—and hence stabilise the protein fold.

This suggests one potential way in which the fold of the mutant WD40-repeat may be retained. A second plausible alternative, is that another, more distant part of the PALB2 protein, can adopt a similar beta-strand conformation to that of 7D, and again stabilise the fold.

Although the in-frame deletion clearly affects the overall stability of the truncated protein in patient cells, is it nevertheless still detectable, albeit at a reduced level.

The mutant form of the protein can also be transiently expressed in U2OS cells, but again is unstable compared with full length PALB2.

Loss of beta-strand 7D, like the Y1183Ter mutation, might be expected to affect closure of the WD40-repeat and lead to protein mis-folding and targeted degradation. However, the mutant protein is clearly functional, as demonstrated by its ability to interact with BRCA2 (Fig 6A) and by facilitating Rad51 recruitment to foci.

## Discussion

Here we describe an unusual family in which the affected individuals have biallelic mutations in *PALB2*, yet do not display the clinical features typically associated with the disease. We propose that the mild clinical phenotype exhibited by the affected patients in this family results from the presence of an expressed mutant PALB2 protein that retains a sufficient level of WT activity.

PALB2 functions as a molecular bridge linking BRCA1-containing DNA repair complexes with the core HR machinery consisting of BRCA2, Rad51 and the Rad51 paralogs. The C-terminal WD40 domain of PALB2 is the major binding site for BRCA2 and as a consequence is essential for the formation of Rad51 foci in response to DNA damage. The N-terminal coiled-coil domain of PALB2 is required for its ability to interact with BRCA1 [3,4]. However, the functional relevance of this interaction is unclear. It has been reported that BRCA1 targets the PALB2-BRCA2 complex to sites of DNA damage. Whether this occurs directly through its ability to bind PALB2, or indirectly via its role in promoting DNA end-resection, is not known. Unlike loss of BRCA2, compromising BRCA1 expression does not completely abrogate the ability of PALB2 to relocalise to sites of DNA damage or promote Rad51 nucleofilament formation, suggesting that this interaction may be required to increase the efficiency of HR. Despite this, there is conflicting evidence as to whether the BRCA1-PALB2 interaction is actually required for DNA repair or not. Simhadri et al, [24] reported that an engineered triple point mutation in the murine *PALB2* gene that reduced its ability to bind BRCA1 gave rise to a mild DNA repair defect. In contrast Xia et al, [15] showed that a C-terminally truncated PALB2 that still retains its BRCA1 binding site could not complement the DNA repair defects exhibited by a FA-N patient-derived cell line. Based on these observations and the fact that we could co-precipitate the T839_K862del but not the Q559RfsTer2 patient-associated mutant PALB2 protein with BRCA2, we suggest that the T839_K862del mutant *PALB2* allele retained sufficient function to alleviate the clinical symptoms associated with a PALB2 deficiency. Structural modelling of this hypomorphic patient mutation indicated that, whilst the loss of residues Thr839-Lys862 located proximal to a small hydrophobic pocket within blade 7 of the WD40 repeat domain is likely to destabilise the protein due to loss of several critical hydrogen bonds, repositioning of upstream hydrophobic residues Cys828, His832 and Val836 may in part compensate for the loss of the missing amino acids and provide some increased stability of the mutant protein (Fig 6). As a consequence, expression of this mutant protein, albeit at reduced levels, may be sufficient to alleviate the clinical symptoms and cellular defects exhibited by the affected patients.

Interestingly, cells derived from the affected patients from this family do not form Rad51 foci in response to either DNA DSBs or cross-links. In contrast, following either IR or MMC exposure Rad51 foci were observed in U2OS cells that expressed the exon 6 deleted T839_K862del mutant PALB2. Therefore, the T839_K862del mutant PALB2 does retain residual function, but it is not expressed at levels sufficient to result in detectable Rad51 foci in patient cells. Rather, it appears that Rad51 is recruited to sites of DNA damage in these patient cells at a level that is below detection by standard immunofluorescence.

The presence of the p.Thr839_Lys862del mutant PALB2 impacts on the ability of the cell to repair DNA damage by HR as shown by the large increase in G2 chromosome damage following IR exposure. The disparity between the IR-induced chromosomal breakage and the colony survival analysis of the affected patient cells is readily explained. The chromosomal analysis assay, described here, predominantly measures G2 phase DSB repair, which will detect defects in both NHEJ and HR. In contrast, the colony survival assay will primarily detect abnormalities in the NHEJ pathway since the DNA damage is induced in an asynchronous culture in which the vast majority of the fibroblasts will be in G1 phase of the cell cycle. Absence of increased radiosensitivity by colony forming assay has been observed in FA-D1 cells [25], although a chromosome assay of FA-D1 lymphocytes exposed at G2 showed them to be very radiosensitive. A similar insensitivity to IR by colony forming ability, but clear increased sensitivity to chromosome damage following G2 irradiation, has also been observed in both fibroblasts and lymphoblasts from patients with Cornelia de Lange Syndrome [26] with a defect in sister chromatid cohesion. Interestingly, our observations, which are consistent with deficient DNA DSB repair as a result of a defect in HR in these hypomorphic *PALB2* patients, using the same endpoint (Table 1) show an effect greater than seen in classical ataxia telangiectasia patients. The G2 chromosomal radiosensitivity of our patients with hypomorphic *PALB2* mutations is also greater than seen in Fanconi patients with likely mutations in the FA core complex proteins [27].

In contrast, following exposure to MMC the colony forming ability of fibroblasts from patients II-4 and II-5, while impaired compared with normal, appeared not to be as deficient as cells from core complex protein Fanconi patients, PALB2 patients [15,16] or FA-D1 patients [25,26]. Published data for chromosomal sensitivity to MMC does not appear to be available for FA-D1 or FA-N patient lymphocytes compared with core complex patients. We assume that the chromosomal sensitivity to MMC of our hypomorphic *PALB2* patient was less than that of a null patient. While null *PALB2* (and BRCA2) patients have a defect in both DNA double strand break repair and also the resolution of DNA crosslinks leading to their profound clinical presentation, the presence of the hypomorphic *PALB2* mutation alleviates this considerably.

The clinical features of the two siblings (II-4 and II-5) shown to have the biallelic *PALB2* mutations included abnormally small size, dysmorphic facies and lymphoid tumour development. It is less clear whether the learning difficulty and speech impairment are part of the *PALB2* syndrome in this family, because of their occurrence in other family members without biallelic mutation of *PALB2*. Most strikingly, these sisters did not present in infancy with the much more severe developmental features and embryonal type tumours associated with biallelic truncating mutations of *PALB2* or, indeed, biallelic truncating mutation of *BRCA2*. However, the sisters shared the Fanconi anaemia patient characteristic of small size. The absence of radial aplasia or abnormalities of the thumbs would not preclude a diagnosis of FA, as not all FA patients show these features. Unlike typical Fanconi anaemia patients, however, 80% of whom have a mutation in a core Fanconi anaemia core protein/gene, they did not have aplastic anaemia, a common presentation of FA between ages 5–10 years. Therefore, although some milder clinical features of FA were present, the phenotype of the affected sisters was clearly distinct from either typical PALB2 (FA-N) patients or typical FA.

A particularly important aspect of the clinical phenotype of the patients with a *PALB2* defect described in this study is that both affected individuals developed B cell non-Hodgkin lymphoma (B-NHL). This tumour type clearly differs from the embryonal-type tumours that normally develop in FA-N or FA-D1 patients. The occurrence of B-NHL in this family could be specific to the particular inherited *PALB2* mutation or could arise as a consequence of the slightly older age of the affected patients in this family as compared to most classical FA-N patients. Nevertheless, the presence of a lymphoid tumour in both affected individuals does raise the interesting possibility that *PALB2* may represent a novel tumour suppressor of lymphoid malignancies in a manner similar to *ATM* and *NBN*.

## Methods

### Cell culture

Epstein-Barr virus (EBV) transformed lymphoblastoid cell lines (LCLs) were established from the younger sister (II-5), both parents (I-1 and I-2) and siblings II-3, II-6 and II-7) (Fig 1) as well as control lymphocytes. LCLs were cultured in RPMI 1640 medium (Sigma-Aldrich, Irvine, UK) supplemented with 10% foetal calf serum (FCS). Fibroblast cultures were also established from skin biopsies from both affected patients II-4 & II-5 and cells grown in DMEM with 10%FCS.

### Assays for γ-irradiation and mitomycin C sensitivity

For colony forming assays, serial dilutions of primary skin fibroblasts from a normal individual, the two affected individuals (II-4 & II-5) in the family under investigation and TERT immortalised fibroblasts from an ataxia telangiectasia patient were irradiated with 1 to 5Gy of γ rays. Patient, normal control and Fanconi Anaemia fibroblasts were exposed for 1h to mitomycin C at concentrations of 0.05–0.40μgml^-1^. Treated fibroblasts were seeded on to a lethally irradiated (35Gy) feeder layer of normal fibroblasts (6x10^4^ cells per 9.0cm dish) in DMEM (Sigma Aldrich) with 10% FCS. Cells were plated in quadruplicate and incubated in 5% CO_2_ at 37°C for 18–21 days with medium changing. The medium was removed, colonies fixed with formaldehyde and stained with 0.5% Methylene Blue (Fisher Scientific, Loughborough, UK). The number of colonies on each plate was counted and percentage survival calculated.

To assess chromosomal radiosensitivity, whole blood cultures in RPMI 1640 + FCS were stimulated with PHA for 72h and exposed to 1Gy ^137^Cs γ-rays 4h before harvesting. Colcemid was added 1h prior to harvesting. Chromosome spreads were made using standard methods and chromosome damage analysed by light microscopy. For chromosomal sensitivity to MMC, whole blood cultures were exposed to either 0.01 or 0.05μgml^-1^ for 30 minutes, washed out and cells harvested 48h later, again with colcemid added 1h prior to harvesting.

### Immunofluorescence

Cells seeded on to coverslips were permeabilized in CSK100 buffer (10 mM PIPES, 20 mM NaCl, 3 mM MgCl_2_, 300 mM sucrose, 0.5% Triton X-100) for 5 min at 4°C and then fixed in 3.6% PFA/PBS for 10 min at 4°C. Coverslips were blocked for 1h in 10% FCS in PBS, incubated for 1h at room temperature with the primary antibody diluted in 2% FCS, washed three times in PBS, and then incubated for an additional 1h with the secondary antibody. Coverslips were washed again three times in PBS and then mounted onto a microscope slide with Vectashield containing DAPI (Vector Laboratories, Burlingame, CA). Primary antibodies used were anti-γH2AX (05–636; Merck Millipore), anti-Rad51 (PC130; Calbiochem), anti-BRCA1 (sc-6954; Santa Cruz Biotechnology), anti-MDC1 (made by G. Stewart), anti-53BP1 (NB100-904; Novus Biologicals), and anti-FANCD2 (sc-20022; Santa Cruz) and anti-RPA2 (NA18; Calbiochem). All secondary antibodies were either Alexa Fluor 488 or Alexa Fluor 594 coupled and purchased from Invitrogen (Carlsbad, CA). Fluorescence images were taken using a Nikon E600 Eclipse microscope 333 equipped with a 60X oil lens, and images were acquired and analysed using Volocity Software 334 v4.1 (Improvision).

### DNA sequence analysis

DNA sequencing was performed on PCR products using the Applied Biosystems BigDye Terminator v3.1 Cycle Sequencing Kit (Part No. 4336917). Sequence analysis of purified products of sequencing reactions was performed using an Applied Biosystems 3500xL Genetic Analyzer. PALB2 sequence variations were designated according to the reference genomic (NG_007406.1), mRNA (NM_024675.3) and protein (NP_078951.2) sequences.

### Immunoblotting for PALB2 expression and ATM kinase activity assays

To analyse the level of PALB2 expression in LCLs, cell pellets were resuspended in UTB buffer (8M urea, 50mM Tris pH 7.5, 150mM β-mercaptoethanol) and lysed on ice by sonication. 50μg of lysate was separated by SDS-polyacrylamide gel electrophoresis and the proteins transferred to nitrocellulose membrane (Pierce). Nitrocellulose strips were subjected to immunoblotting and protein bands visualised using the enhanced chemiluminescence (ECL) system and exposure to Hyper-film (GE Healthcare). PALB2 antibodies used for immunoblotting were rabbit polyclonals A301-246A (Bethyl Laboratories) that recognises an epitope between residues 200–250 of PALB2 and A301-247A that recognises an epitope between residues 675–725 (see Fig 5). Other antibodies used were anti-BRCA2 (OP95; Calbiochem), anti-BRCA1 (OP92; Calbiochem), anti-Rad51 (SC-8349; Santa Cruz), anti-Aprataxin (made in house) and anti-FLAG (F1804; Sigma-Aldrich).

To analyse ATM signalling cells were either mock-irradiated or exposed to 5Gy ionising radiation and harvested after 30 minutes. Antibodies used for immunoblotting were: ATM 11G12 (made in house), anti-phospho ATM (S1981) (AF1655; R&D Systems), anti-SMC1 (A300-055A; Bethyl Laboratories), anti-phospho SMC1 (S966) (A300-050A; Bethyl), anti-KAP-1 (A300-274A; Bethyl), anti-phospho KAP-1 (S824) (A300-767A; Bethyl), anti-NBN (ab23996; Abcam), anti-phospho NBN (S343) (ab47272; Abcam), anti-phospho CHK2 (T68) (2661; Cell Signaling Technology, New England Biolabs), anti-CREB (48H2; Cell Signaling), anti-phospho CREB (S121) (NB100-410; Novus Biologicals), anti-γH2AX (05–636; Merck Millipore), anti-H2A (07–146; Merck Millipore), anti-phospho BRCA1 (S1423) (Bethyl), anti-BRCA1 (OP92; Calbiochem), anti-Chk2 (gift from Dr S. Elledge), anti-53BP1 (NB100-904; Novus Biologicals) and anti-phospho 53BP1 (S1778) (2675; Cell Signaling Technology).

### Creation of stable Flp-In/T-Rex U2OS cell lines

Isogenic U2OS cell lines inducibly expressing either WT or mutant FLAG-tagged PALB2 were created using the Flp-In/T-Rex system. WT PALB2, or a series of PALB2 mutants were cloned into the pcDNA5/FRT/TO plasmid containing an N-terminal FLAG tag and then transfected into U2OS cells containing a single FRT recombination site. Transfected cells were selected with hygromycin and blasticidin and then cloned. Expression of the inducible FLAG-tagged PALB2 protein in each of the cell clones was determined by Western blotting. The PALB2 mutants generated in this study were a revertant *PALB2* exon 4 deletion (Exon4delRev) and a FA-N patient-derived mutation Y551Ter, both previously described by Xia et al (15) and both exon 6 deletion (p.T839_K862del) (c. 2586+1G>A; p.Thr839_Lys862del) and Q559RfsTer2 (c.1676_1677delAAinsG; p.Gln559ArgfsTer2) identified in our patients.

### Knockdown of endogenous PALB2 using siRNA

A PALB2 siRNA (GGAGAAUAUCUGAAUGACAdTdT) directed against the 3’ UTR region of PALB2 mRNA [4] was used to knockdown endogenous PALB2. Cells were transfected with siRNA using Lipofectamine RNAiMAX Reagent (Invitrogen) according to the manufacturers protocol. Four hours post-transfection 1μg/ml doxycycline was added to the cells to induce exogenous PALB2 protein expression. The PALB2 cDNA sequences cloned in the pcDNA5-FLAG/FRT/TO vector did not contain 3’-UTR sequences and transcripts from these constructs were not targeted by the siRNA. Cells were harvested 48h post-transfection. The efficiency of PALB2 knockdown was determined by Western blotting.

### Immunoprecipitation

Immunoprecipitations were performed using a protocol published previously [28].

## Supporting Information

S1 Fig Measurement of ATM signaling in cells from affected siblings. ATM signaling assay on fibroblasts from affected siblings measured by western blotting for both II-4 & II-5 reveals normal ATM signaling as indicated by phosphorylation of ATM targets.
(PDF)

S2 Fig Intranuclear redistribution of DNA repair proteins in affected siblings. Following exposure to IR or mitomycin C fibroblasts from patients II-4 and II-5 were able to relocalise MDC1, 53BP1, BRCA1, FANCD2 and RPA2 to sites of DNA DSBs marked by γH2AX foci in a manner similar to the normal fibroblast cell line. Fluorescence images were taken using a Nikon E600 Eclipse microscope 333 equipped with a 60X oil lens, and images were acquired and analysed using Volocity Software 334 v4.1 (Improvision).
(PDF)

S3 Fig Reduced level of BRCA2 in affected sibling. Western blot showing a reduced level of BRCA2 and the absence of full length PALB2 in cells from affected patient II-5 (performed in duplicate). Loading control is aprataxin.
(PDF)

S4 Fig Doxycycline induced expression of the different PALB2 mutant proteins in U2OS cells. Western blot showing doxycycline induced expression of FLAG-tagged WT PALB2 protein and each of the mutant PALB2 proteins, Y551Ter, Q559RfsTer2, T839_K862del and Exon4delRev following siRNA knockdown of endogenous PALB2 in the same cell cultures as used for the Rad51 immunofluorescence (Fig 7).
(PDF)
