# Supplementary figures and images for: A Hypomorphic PALB2 Allele Gives Rise to an Unusual Form of FA-N Associated with Lymphoid Tumour Development
Source: PLoS Genet. 2016 Mar 18;12(3):e1005945. doi: 10.1371/journal.pgen.1005945 (PMC4798644; doi:10.1371/journal.pgen.1005945)

**Figure S1**

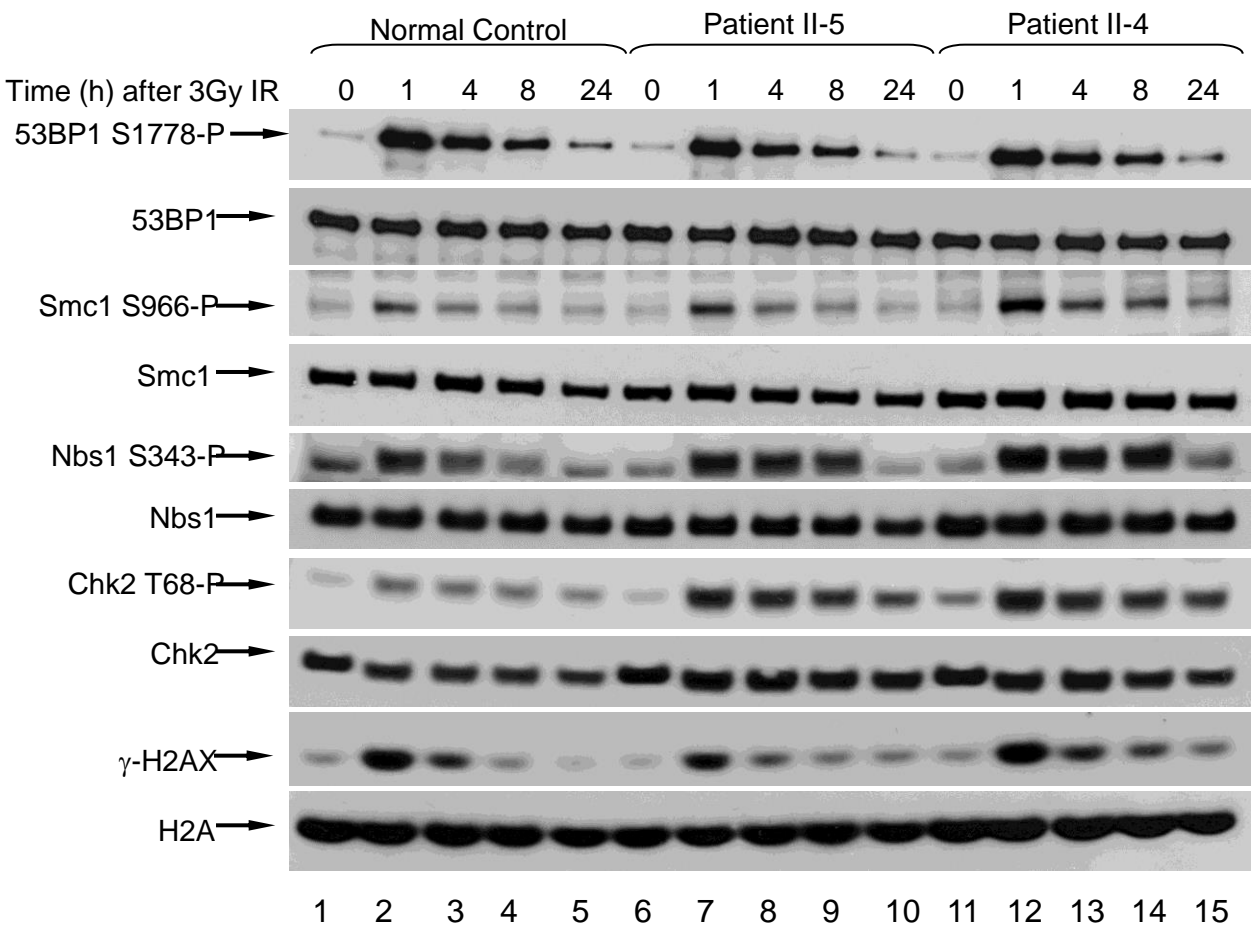

Supplement: S1 Fig — ATM signaling assay on fibroblasts from affected siblings measured by western blotting for both II-4 & II-5 reveals normal ATM signaling as indicated by phosphorylation of ATM targets. (PDF) [file pgen.1005945.s001.pdf]

**Figure S2**

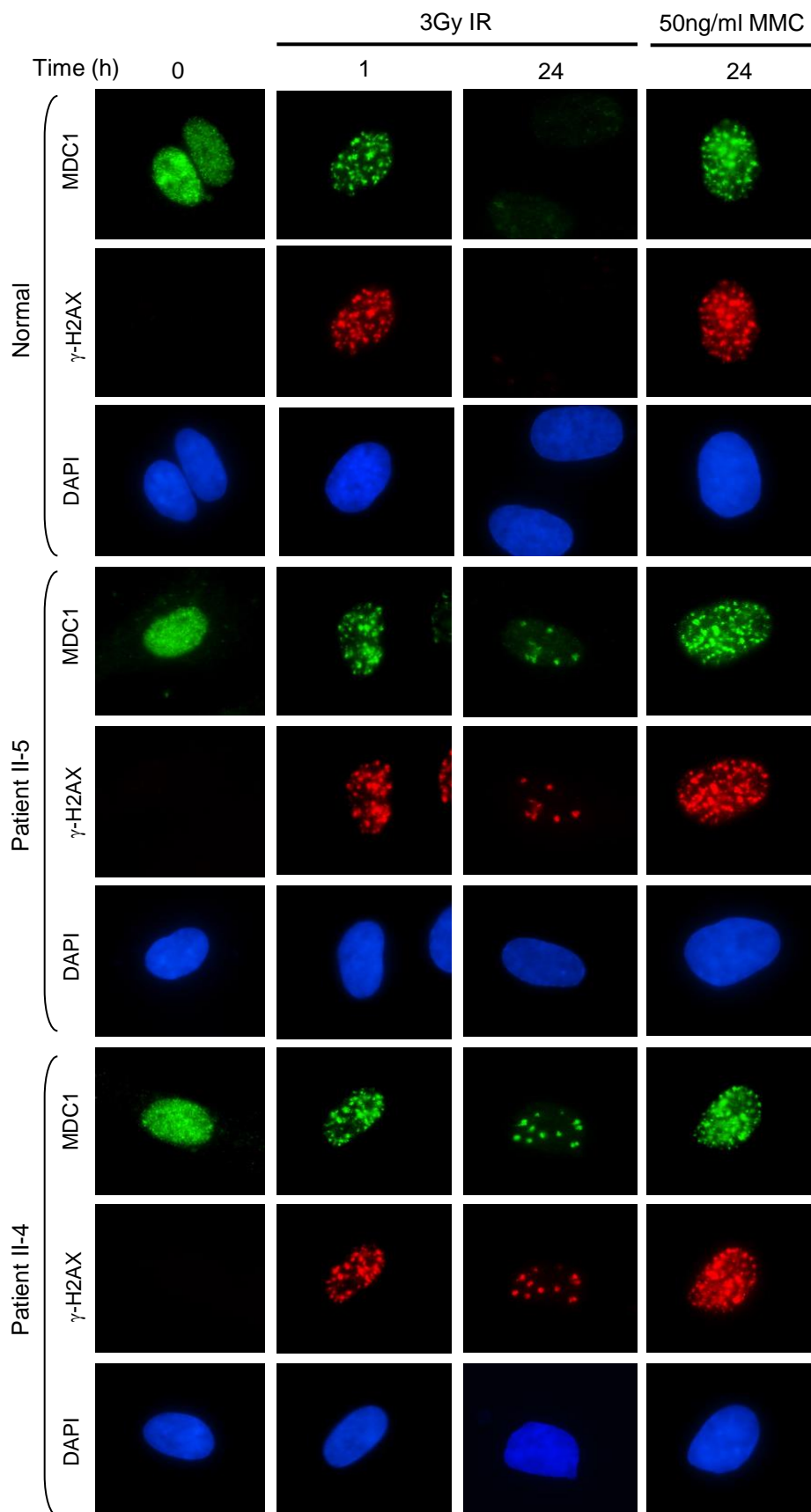

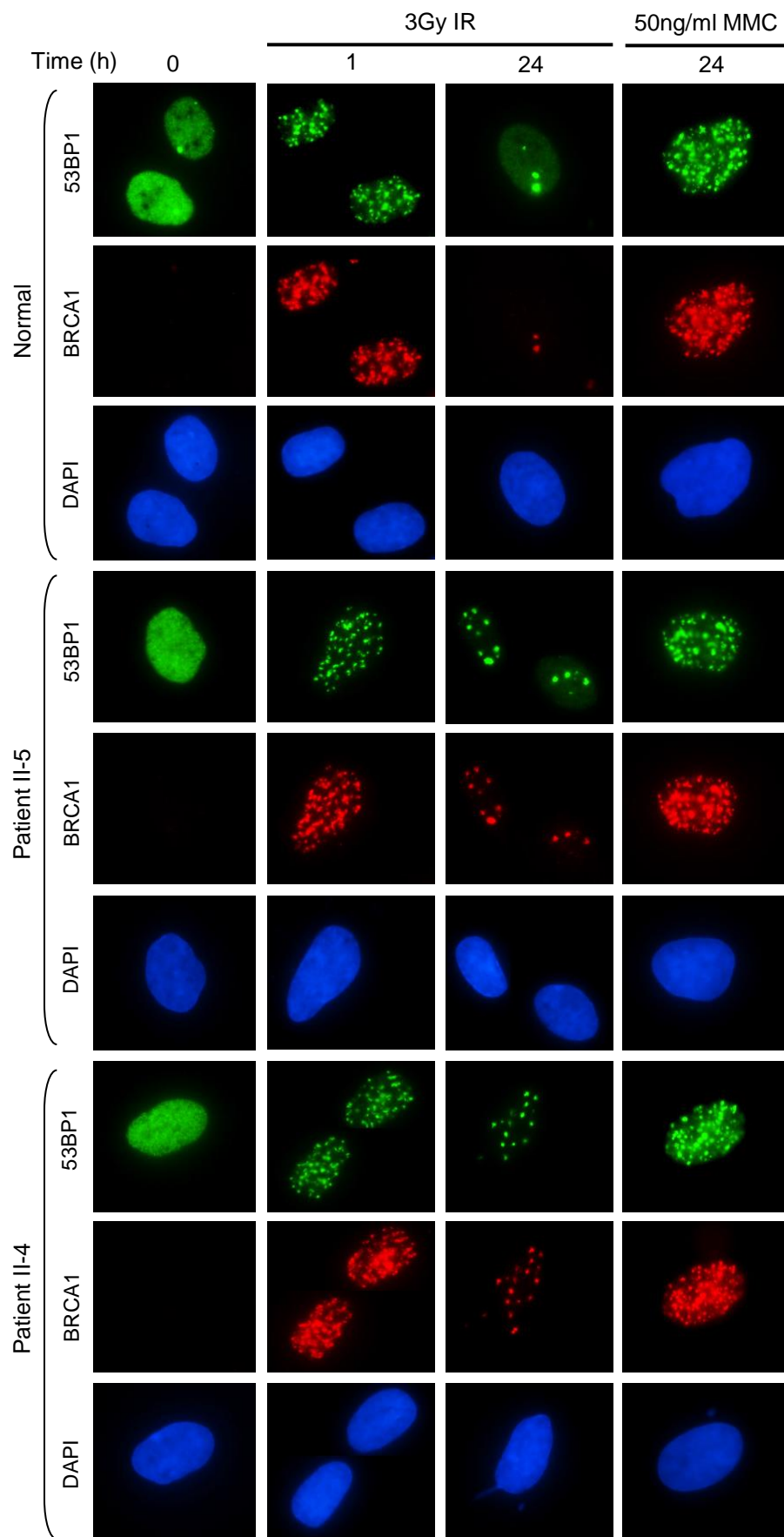

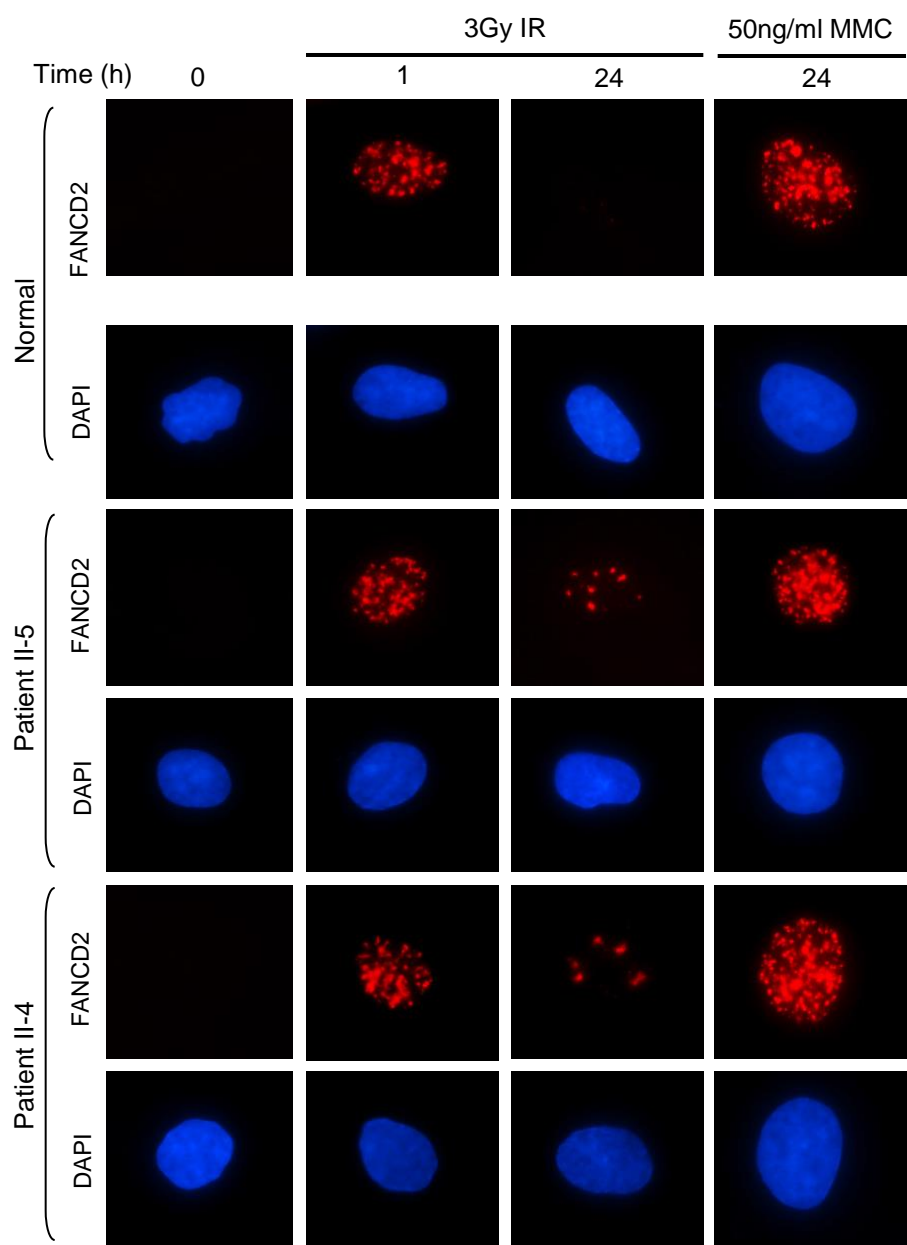

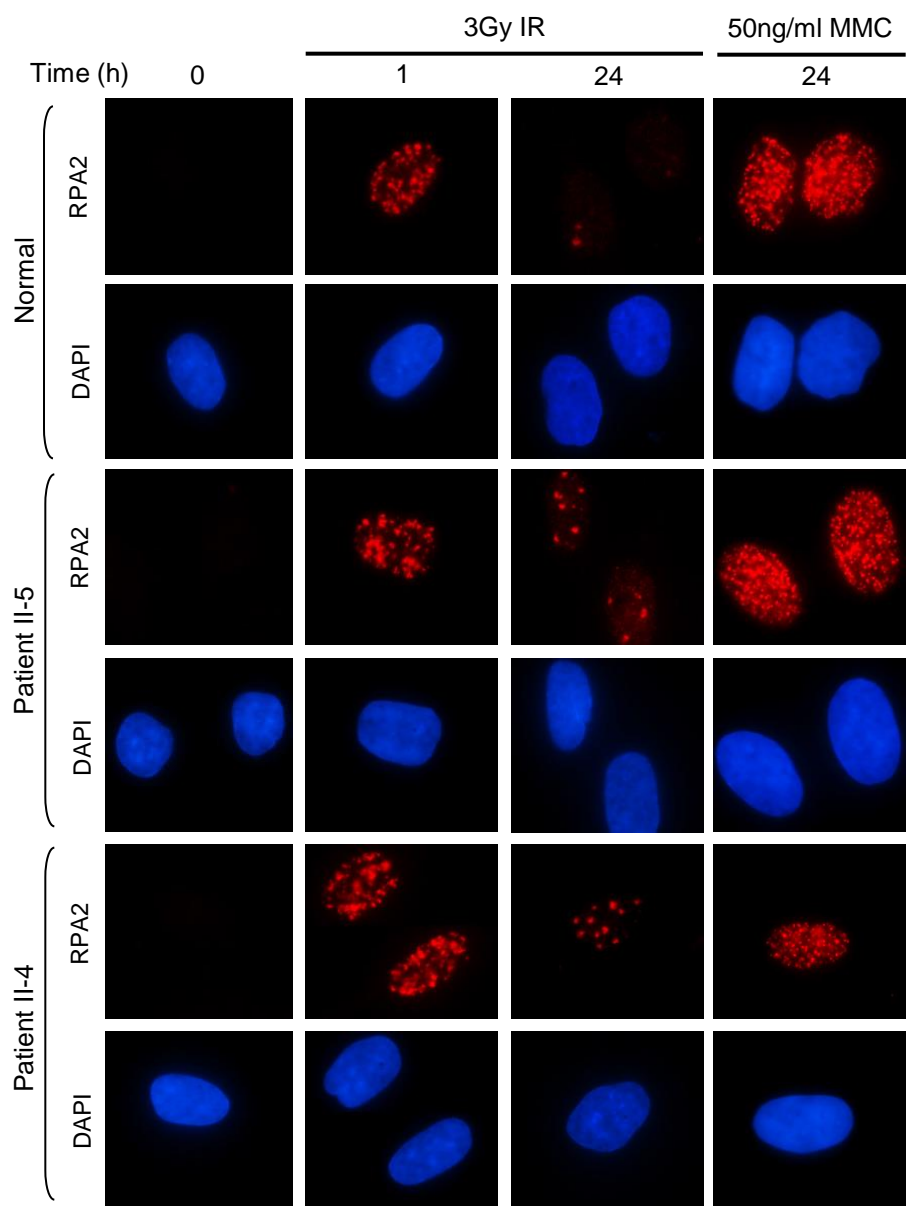

Supplement: S2 Fig — Following exposure to IR or mitomycin C fibroblasts from patients II-4 and II-5 were able to relocalise MDC1, 53BP1, BRCA1, FANCD2 and RPA2 to sites of DNA DSBs marked by γH2AX foci in a manner similar to the normal fibroblast cell line. Fluorescence images were taken using a Nikon E600 Eclipse microscope 333 equipped with a 60X oil lens, and images were acquired and analysed using Volocity Software 334 v4.1 (Improvision). (PDF) [file pgen.1005945.s002.pdf]

### Figure S3

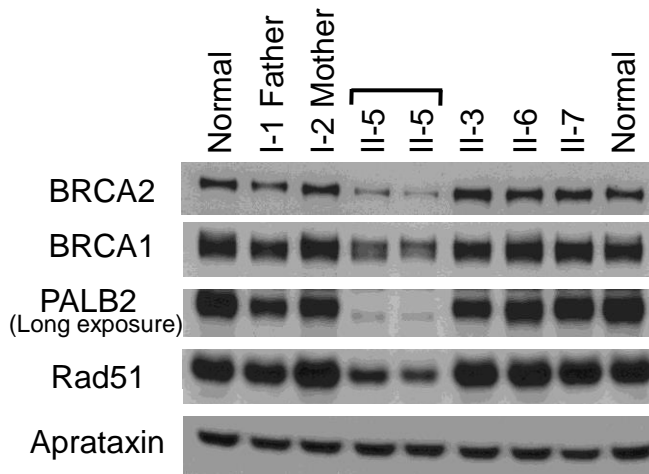

Supplement: S3 Fig — Western blot showing a reduced level of BRCA2 and the absence of full length PALB2 in cells from affected patient II-5 (performed in duplicate). Loading control is aprataxin. (PDF) [file pgen.1005945.s003.pdf]

**Figure S4**

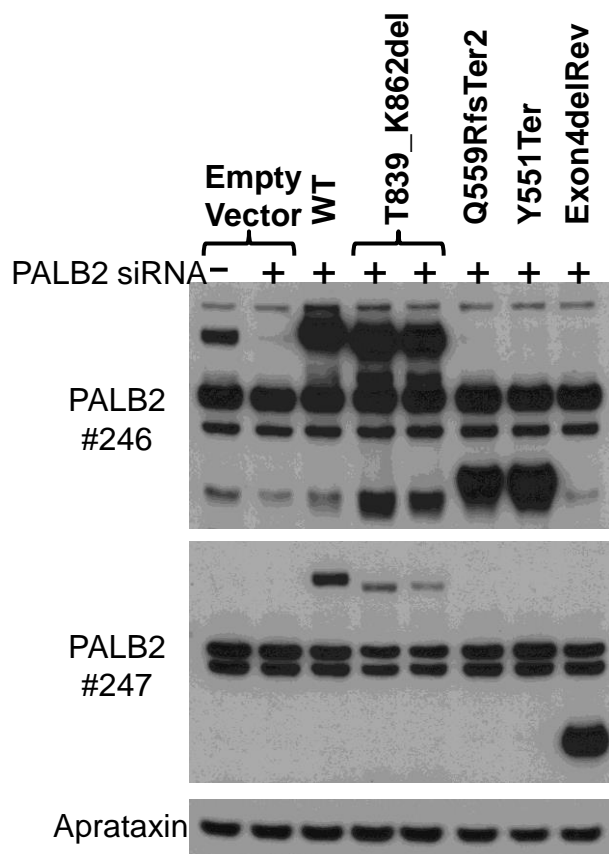

Supplement: S4 Fig — Western blot showing doxycycline induced expression of FLAG-tagged WT PALB2 protein and each of the mutant PALB2 proteins, Y551Ter, Q559RfsTer2, T839_K862del and Exon4delRev following siRNA knockdown of endogenous PALB2 in the same cell cultures as used for the Rad51 immunofluorescence (Fig 7). (PDF) [file pgen.1005945.s004.pdf]
